# Indoxyl Sulfate and Incident Peripheral Artery Disease in Hemodialysis Patients

**DOI:** 10.3390/toxins12110696

**Published:** 2020-11-02

**Authors:** Ting-Yun Lin, Hsin-Hua Chou, Hsuan-Li Huang, Szu-Chun Hung

**Affiliations:** 1 Division of Nephrology, Taipei Tzu Chi Hospital, Buddhist Tzu Chi Medical Foundation, and School of Medicine, Tzu Chi University, Hualien 970, Taiwan; water_h2o_6@hotmail.com; 2 Division of Cardiology, Taipei Tzu Chi Hospital, Buddhist Tzu Chi Medical Foundation, and School of Medicine, Tzu Chi University, Hualien 970, Taiwan; chouhhtw@gmail.com

**Keywords:** chronic kidney disease, hemodialysis, indoxyl sulfate, major adverse cardiovascular events, mortality, peripheral artery disease

## Abstract

Peripheral artery disease (PAD) is highly prevalent among patients with chronic kidney disease (CKD) and portends a very poor prognosis. Indoxyl sulfate has been shown to induce atherothrombosis and impaired neovascularization in uremic mice. However, there is no clinical evidence regarding the role of indoxyl sulfate in PAD associated with CKD. We examined associations between indoxyl sulfate and incident symptomatic lower extremity PAD events as well as major adverse cardiovascular events (MACE) and all-cause mortality using Cox proportional hazards models in a prospective cohort of 200 hemodialysis patients free of PAD at baseline. Patients were considered as having PAD if they developed PAD symptoms confirmed by an ankle-brachial index with waveforms, duplex ultrasound or angiography, and/or major adverse limb events including revascularization and amputation. During a median follow-up of 6.5 years, 37 patients (18.5%) experienced incident symptomatic PAD. MACE occurred in 52 patients, and a total of 85 patients died. After adjusting for traditional risk factors for PAD, including age, current smoking, diabetes, and cardiovascular disease, indoxyl sulfate was significantly associated with the risk of PAD (hazard ratio (HR), 1.19 for every 10-μg/mL increase in indoxyl sulfate; 95% confidence interval (CI), 1.05–1.35). However, indoxyl sulfate was not associated with risk of MACE (HR, 1.00; 95% CI, 0.90–1.12) or death from any cause (HR, 0.98; 95% CI, 0.90–1.07). Indoxyl sulfate was associated with incident symptomatic PAD but not with MACE or all-cause mortality, suggesting that indoxyl sulfate toxicity may be unique to PAD among hemodialysis patients.

## 1. Introduction

Patients with chronic kidney disease (CKD) are at a higher risk of developing peripheral artery disease (PAD) and its adverse outcomes than the general population. Data from the 2018 US Renal Data System annual data report showed that the prevalence of PAD was 25.2% among patients aged 66 and older who had CKD, compared to 9.7% among those who did not have CKD [1]. The risk of PAD is markedly increased in patients with end-stage kidney disease (ESKD) requiring dialysis. Among hemodialysis and peritoneal dialysis patients aged 22 and older, the prevalence of PAD was 37.4% and 25.0%, respectively [1]. The presence of CKD also significantly worsened both limb and mortality outcomes. Despite improvements, the rate of lower extremity amputation remained more than 20-fold higher among patients on dialysis than among the general population [2]. In addition, nearly half of all patients who have undergone lower extremity amputation died within the first year after the procedure [2].

Identifying factors that are associated with PAD is important in the care of patients with CKD [3,4]. Traditional cardiovascular risk factors for the general population, such as diabetes mellitus, hypertension, and dyslipidemia, are more common in patients with CKD but cannot fully explain the increased risk and adverse consequences of PAD in this population [5]. CKD is an independent risk factor for incident PAD, with risk increasing as kidney function worsens [6]. Beyond the traditional risk factors, the accumulation of uremic toxins in CKD may contribute to the pathogenesis of PAD. Indoxyl sulfate, derived from the intestinal microbial metabolism of dietary tryptophan, is among the most well studied uremic toxins. Indoxyl sulfate circulates mostly bound to albumin and is poorly removed by hemodialysis. We have shown that indoxyl sulfate suppresses endothelial progenitor cell-mediated neovascularization in uremic mice with experimental unilateral hindlimb ischemia [7]. We hypothesized that the observed impaired neovascularization could explain the increased risk of PAD in CKD. In the present study, we sought to determine the association of indoxyl sulfate levels with incident symptomatic lower extremity PAD events as well as major adverse cardiovascular events (MACE) and all-cause mortality among patients with ESKD who received hemodialysis.

## 2. Results

### 2.1. Baseline Characteristics of Patients

**Figure 1:**
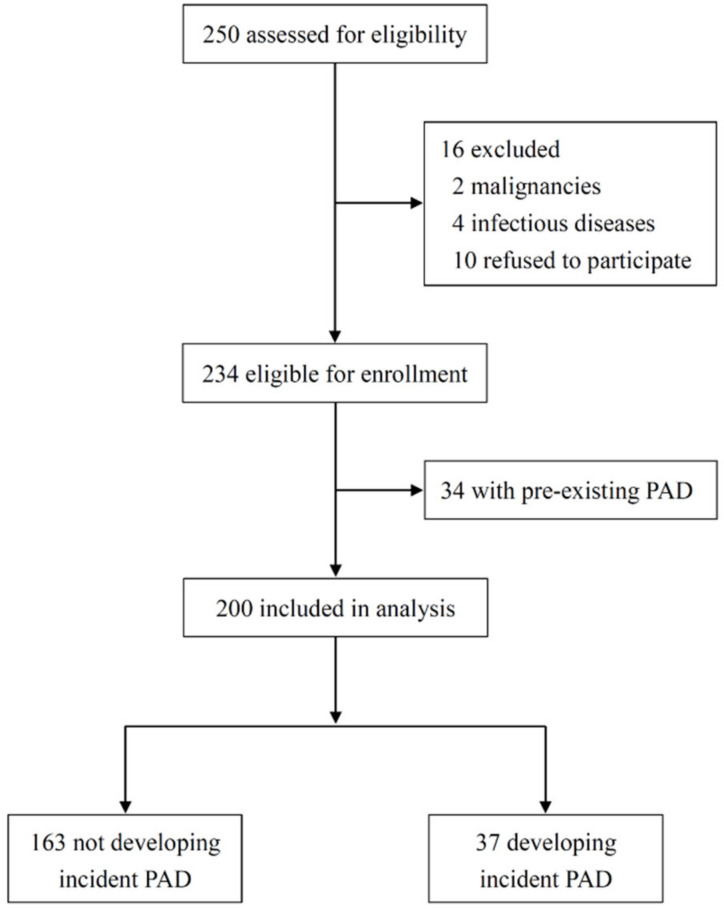
Patient flow diagram. PAD, peripheral artery disease.

The original number of participants included 250 patients, of which 2 were excluded due to active malignancy, 4 were excluded for active infectious disease, and 10 refused to participate (Figure 1). Table 1 summarizes the baseline characteristics of 234 patients divided by PAD status. The mean age was 63 ± 13 years, 54.7% were male, and the median dialysis vintage was 2.4 (interquartile range, 1.4–3.7) years. Overall, 44.0% (*n* = 103) had diabetes, 32.5% (*n* = 76) had cardiovascular disease (CVD), and 14.5% (*n* = 34) had pre-existing PAD. Patients with PAD were older and more likely to have diabetes and concomitant CVD. As expected, ankle-brachial index (ABI) was significantly lower in this group, and prescription rates of antiplatelets and statins were significantly higher. Notably, major traditional risk factors for PAD, including male sex, smoking history, body mass index (BMI), systolic blood pressure (BP), triglycerides, and the total cholesterol (TC): high-density lipoprotein cholesterol (HDL-C) ratio, did not differ between patients with and without PAD. There were also no significant differences in other risk factors for PAD reported in the CKD population, such as dialysis vintage, plasma phosphate, intact parathyroid hormone (PTH), and C-reactive protein (CRP) levels between the 2 groups. A total of 200 patients who were free of PAD were followed for clinical outcomes from 1 September 2008, to 30 June 2020.

### 2.2. Indoxyl Sulfate and Incident PAD

During a median follow-up time of 6.5 years, 37 patients (18.5%) experienced a first PAD event. As shown in Table 2, patients with incident PAD were older and had a higher prevalence of diabetes and CVD. Despite similar BP measurements, lipid profiles, glycemic control, serum calcium and phosphate levels, and CRP, patients who developed incident PAD had significantly higher indoxyl sulfate concentrations. Table 3 shows associations of risk factors and indoxyl sulfate with incident PAD from multivariable Cox regression analyses. Patients were stratified according to tertiles of serum indoxyl sulfate concentrations. Indoxyl sulfate was significantly associated with incident PAD in fully adjusted models (hazard ratio (HR) for the upper versus lower tertile, 3.20; 95% confidence interval (CI), 1.26–8.12; *p* for trend = 0.013). When indoxyl sulfate was analyzed as a continuous variable, the findings were similar. For every 10-μg/mL increase in indoxyl sulfate, the hazards of incident PAD events increased by 19% (*p* = 0.006). In addition, the strong association was also identified between other risk factors and incident PAD, including age, diabetes, and the presence of CVD.

**Figure 2:**
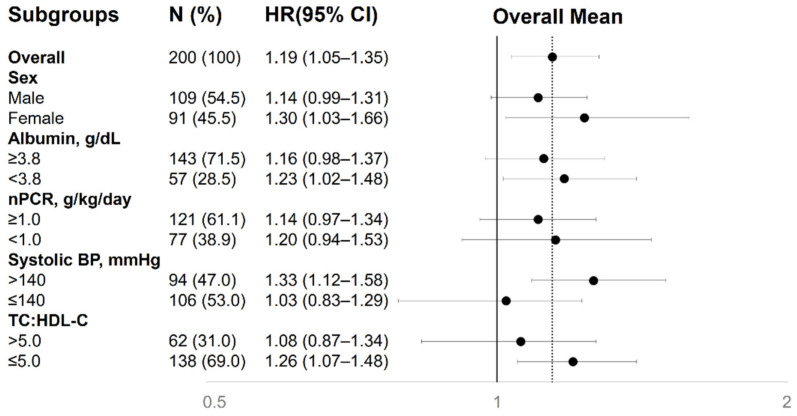
Association between serum indoxyl sulfate and incident PAD in different clinical subgroups. The fully adjusted hazard ratios are shown as the fold of risk of incident PAD per-10 μg/mL increase in indoxyl sulfate. BP, blood pressure; CI, confidence interval; HR, hazard ratio; nPCR, normalized protein catabolic rate; TC:HDL-C, total cholesterol:high-density lipoprotein ratio.

We performed subgroup analysis to explore the association between indoxyl sulfate and incident PAD in the cohort stratified by sex, albumin (≥3.8 vs. <3.8 g/dL), normalized protein nitrogen appearance (nPCR) (≥1.0 vs. <1.0 g/kg/day), systolic BP (>140 vs. ≤140 mmHg), and the TC:HDL-C ratio (>5.0 vs. ≤5.0) (Figure 2). The increased risk of PAD associated with indoxyl sulfate was consistent across all clinically relevant subgroups (*p* for interaction >0.05 for all).

### 2.3. Indoxyl Sulfate and MACE or All-cause Mortality

There were 52 cases of MACE, including nonfatal myocardial infarction (*n* = 9), nonfatal stroke (*n* = 15) and cardiovascular death (*n* = 28). A total of 85 patients died from cardiovascular (*n* = 31) and noncardiovascular (*n* = 54) events. Cardiovascular deaths included deaths from ischemic heart disease (*n* = 2), cerebrovascular disease (*n* = 3), congestive heart failure (*n* = 7), arrhythmia (*n* = 2), and sudden cardiac deaths (*n* = 17). Noncardiovascular causes consisted of infectious disease (*n* = 33), cancer (*n* = 12), suicide (*n* = 2), and others (*n* = 7). Table 4 shows the associations of each of the known risk factors with PAD, MACE, and all-cause mortality after adjusting for age, current smoking, diabetes, and CVD. Among these risk factors, only indoxyl sulfate was significantly associated with incident PAD. In contrast, no significant associations were seen between indoxyl sulfate and MACE or all-cause mortality. We found that serum calcium was positively and strongly associated with both MACE (HR, 1.61; 95% CI, 1.15–2.27; *p* < 0.01) and mortality (HR, 1.46; 95% CI, 1.12–1.89; *p* < 0.01). Similar results were seen for MACE per 10-mg/dL increase in low-density lipoprotein cholesterol (LDL-C) (HR, 1.10; 95% CI, 1.00–1.21; *p* < 0.05) and for mortality per 1 unit increase in Ln CRP (HR, 1.31; 95% CI, 1.10–1.56; *p* < 0.01).

## 3. Discussion

Our prospective investigation demonstrated that higher levels of serum indoxyl sulfate were independently associated with a higher risk of incident symptomatic PAD among hemodialysis patients. However, no significant associations between indoxyl sulfate and MACE or all-cause mortality were identified. Our findings have important clinical implications because PAD is highly prevalent in hemodialysis patients and portends a very poor prognosis [8,9,10]. To our knowledge, no prior studies have explored the long-term impact of serum indoxyl sulfate on PAD events in patients with CKD. Most importantly, our results provide valuable insight into the distinct role of indoxyl sulfate in PAD compared with coronary or cerebrovascular atherosclerosis.

An increasing body of experimental data suggests that indoxyl sulfate may contribute to endothelial dysfunction and accelerate atherosclerosis in patients with CKD [11]. Indoxyl sulfate inhibits nitric oxide production, upregulates endothelial expression of adhesion molecules, and enhances leukocyte endothelial interactions by inducing oxidative stress [12,13,14]. Despite a strong scientific rationale for indoxyl sulfate toxicity, clinical data for associations of elevated indoxyl sulfate concentrations with coronary or cerebrovascular atherosclerosis are less robust. Wu et al. investigated the association between total or free indoxyl sulfate with cardiovascular or all-cause mortality in 112 elderly hemodialysis patients [15]. Similar to our results showing a lack of association between indoxyl sulfate and MACE or all-cause mortality, they found that indoxyl sulfate was not associated with outcomes. In the largest sample comprised of 1273 prevalent hemodialysis patients participating in the HEMO study, Shafi et al. determined the association of total indoxyl sulfate with the first cardiovascular event or cardiac death [16]. Overall, there were no associations between the uremic solute and cardiovascular outcomes. However, there were trends of toxicity among patients with lower serum albumin, even though lower serum albumin was associated with lower levels of indoxyl sulfate. In the present study, indoxyl sulfate levels were up to 90-fold higher in hemodialysis patients than in healthy individuals, so a threshold effect may be responsible for the lack of observed associations [17].

It is well established that major risk factors for PAD are not different from those for coronary or cerebral artery disease, but the prognostic importance of these factors may differ in various arterial territories. In general, there is a stronger association of cigarette smoking with PAD, while hypertension and dyslipidemia are more strongly associated with coronary artery disease. A recent study further demonstrated that an atherogenic dyslipidemia profile, including the TC:HDL-C ratio, as well as total and small LDL particle concentrations, appears to be more strongly associated with incident PAD than with coronary artery or cerebrovascular disease in women [18]. In our study, although total indoxyl sulfate shows its limitation as a risk biomarker of MACE or all-cause mortality in hemodialysis patients, it is significantly associated with incident PAD events. Our findings indicate an alternative indoxyl sulfate-related biology in PAD compared with coronary or cerebrovascular atherosclerosis in CKD. Recently, Narula et al. characterized the pathology of PAD in 239 peripheral arteries from 75 patients with critical limb ischemia, of whom 64% had CKD [19]. Unlike the atherosclerotic process in the coronary arterial bed, thrombotic luminal occlusion with insignificant atherosclerosis was frequently observed in patients with critical limb ischemia, suggesting the possibility of thromboembolic disease.

Indoxyl sulfate-induced hypercoagulability may play a role in the pathogenesis of the prothrombotic state in symptomatic PAD. Biologically, indoxyl sulfate increases endothelial expression of tissue factor via aryl hydrocarbon receptor activation [20]. Clinically, both circulating tissue factor concentration and activity are elevated and are positively correlated with plasma indoxyl sulfate in patients with CKD [20]. Tissue factor serves as the primary initiator of the coagulation cascade and is an essential mediator of hemostasis and trigger of thrombosis [21]. By inducing endothelial dysfunction and tissue factor production, indoxyl sulfate favors atherosclerosis and thrombosis, which in turn results in limb ischemia. In the presence of tissue hypoxia, however, indoxyl sulfate further suppresses proangiogenic functions of endothelial progenitor cells, leading to impaired neovascularization and development of symptomatic PAD [22]. The results of our recent study also demonstrated that serum indoxyl sulfate is an independent predictor for dialysis vascular access thrombosis after angioplasty [23]. Thus, the observed association in our study is in agreement with the recognition of increased thrombogenicity elicited by elevated levels of indoxyl sulfate in CKD and possibly represents an effect driven by increased expression of tissue factor. Our findings on the association of indoxyl sulfate with PAD in CKD are not only clinically important but also mechanistically relevant.

Our study has several strengths, including its prospective design in a well-characterized cohort, careful ascertainment of PAD events, and relatively long follow-up period of more than 10 years. However, there are several limitations. First, as is the case for any observational study, we were unable to establish the causality of the relationship between serum indoxyl sulfate and clinical outcomes. Second, patients enrolled in this study were from a single dialysis center; as a result, the findings may not be generalizable to the overall hemodialysis population. Third, serum indoxyl sulfate levels were only measured once at baseline. However, the variability of concentrations of uremic toxins over time within the individual patient might have an impact on the association of indoxyl sulfate with outcomes [24]. Fourth, we used total concentrations of indoxyl sulfate as the outcome predictor rather than free concentrations. Circulating indoxyl sulfate is largely protein-bound. As tissues are exposed to free solutes, free indoxyl sulfate concentrations are presumed to be a better indicator for potential toxicity. However, the free solute levels are also more likely to be influenced by other unmeasured protein-bound uremic toxins that may replace indoxyl sulfate from their binding sites, leading to higher free indoxyl sulfate levels [16,25]. This effect may therefore result in uncontrolled confounding and biased results in a clinical study. Fifth, the use of symptomatic PAD as the primary outcome by definition excluded subclinical cases. However, a substantial number of asymptomatic patients with low ABI never develop clinical symptoms of PAD and the evidence on screening and treatment in these subjects is currently lacking [26]. Sixth, we did not determine other uremic toxins at baseline. Although other uremic toxins may affect clinical outcomes as well, indoxyl sulfate is known to induce the strongest production of reactive oxygen species from endothelial cells [27] and has prothrombotic effects on the endothelium via tissue factor induction, both of which contribute to thrombotic occlusion and symptomatic PAD. For these reasons, indoxyl sulfate may be an appropriate measure for the assessment of uremic toxicity in PAD, but further studies comparing the toxicity of various uremic toxins in ESKD patients are warranted. Finally, given the limited event number, our study was unable to account for multiple confounders in the adjusted analysis.

## 4. Conclusions

Serum indoxyl sulfate levels were independently associated with incident symptomatic PAD events among hemodialysis patients but not with MACE or all-cause mortality. Our findings suggest that measurement of indoxyl sulfate levels might provide a rationale for risk stratification for PAD in patients with ESKD. However, whether serum indoxyl sulfate is a therapeutic target needs further investigation.

## 5. Materials and Methods

### 5.1. Study Design

This prospective cohort study was conducted at the hemodialysis unit of Taipei Tzu Chi Hospital, Taiwan. Patients older than 20 years who had been on hemodialysis for more than 6 months were assessed for eligibility from July to August 2008. Exclusion criteria were inadequacy of dialysis, defined as Kt/V urea <1.2, and conditions of active malignancy or infectious disease. The study protocol was approved by the institutional review board at Taipei Tzu Chi Hospital (98-IRB-001-XD). Written informed consent was obtained from all participants, and the study complied with the Declaration of Helsinki.

For all participants, smoking status and medical history were assessed at the time of study enrollment. Smokers were categorized as current, past, or never smokers. Diabetes was defined by self-reported history or the use of oral antidiabetic agents or insulin. The definition of CVD comprised coronary artery disease, as documented by coronary angiography or a history of myocardial infarction, or a cerebrovascular event. The presence of PAD was defined as a physician diagnosis of lower extremity PAD together with a previous intervention such as angioplasty, stenting, atherectomy, peripheral arterial bypass grafting or amputation, or together with use of cilostazol or antiplatelet therapy. Patients with prevalent PAD at baseline were excluded from further analyses of outcome measures.

### 5.2. Laboratory Measurements

All blood samples were collected from patients who had fasted overnight immediately before the start of a mid-week dialysis session in September 2008. Plasma and serum were separated and kept frozen at −70 °C when not analyzed immediately. Serum biochemical parameters, including glucose, TC, triglycerides, HDL-C, LDL-C, calcium, phosphate, intact PTH, albumin, and CRP, were analyzed by standard laboratory procedures. The adequacy of dialysis was estimated by measuring mid-week urea clearance (Kt/V) using the standard method [28]. Dietary protein intake was estimated by calculating nPCR derived from a two-BUN measurement, single-pool, variable-volume kinetic model [29]. Total indoxyl sulfate (i.e., both free and protein-bound fractions) was obtained via protein precipitation of the serum sample. Indoxyl sulfate levels were measured using a high-performance liquid chromatography–fluorescence method. The analyte was separated by a Luna phenyl-hexyl column (150 × 4.6 mm, 5 μm; Phenomenex, Torrance, CA, USA). The mobile phase consisting of 10 mM phosphate buffer at pH 6.0 and acetonitrile (75:25, volume/volume) was eluted at a flow rate of 1.0 mL/min. The fluorescence detector was set at λex 280 nm/λem 346 nm. BP and the ABI were measured by trained nursing staff before the mid-week hemodialysis session. BP was determined in the nonaccess arm after a 5-min rest while the patient was seated with both feet on the floor using an automated sphygmomanometer. ABI was calculated by taking the systolic BP in the right or left ankle and dividing by the BP in the right or left arm by an automated oscillometric technique (Colin VP-1000; Colin Co., Ltd., Komaki, Japan), and the lowest value was taken for this analysis.

### 5.3. Outcomes

The primary outcome of interest for the present study was incident symptomatic lower extremity PAD in patients free of PAD at baseline. Patients with incident PAD events presented with symptoms of PAD, including typical intermittent claudication, atypical lower extremity symptoms (such as leg numbness, leg weakness, pain or discomfort that begins at rest but worsens with exertion, pain or discomfort that does not stop an individual from walking, and pain or discomfort that begins with exertion but is not alleviated within 10 min of rest), or symptoms of chronic arterial insufficiency (such as resting pain, skin necrosis, ulceration of the foot, skin necrosis, cold temperature of the foot, and reduced capillary refill). The diagnosis of PAD was confirmed with an ABI ≤0.9, an abnormal pulse volume recording waveform, or the presence of arterial stenosis ≥50% on duplex ultrasound [30], computed tomography angiography, or catheter-based radiocontrast angiography and/or hospitalizations for a major adverse limb event, defined as lower extremity revascularization or amputation. Patients were censored at the time of their last contact, kidney transplantation, or death unrelated to a PAD event or at the end of follow-up on 30 June 2020.

The secondary outcomes were MACE and all-cause mortality. MACE included nonfatal myocardial infarction, nonfatal stroke, and cardiovascular death. A trained physician who had no knowledge of the results of the serum indoxyl sulfate measurements independently reviewed all suspected cardiovascular events by examining each medical chart. Patients were censored at the time of their last contact, kidney transplantation, or death due to noncardiovascular causes or at the end of follow-up. Causes of death were ascertained from official death certificates. For all-cause mortality, patients were censored at the time of their last contact, kidney transplantation, or at the end of follow-up.

### 5.4. Statistical Analyses

Data are presented as the mean ± SD for normally distributed continuous variables, median and interquartile range for skewed continuous variables, and frequency and percentage for categorical variables. The baseline characteristics of the subjects with or without pre-existing PAD were compared using a t-test, x^2^ statistics, and the Mann–Whitney U test as appropriate. A multivariable Cox proportional hazards regression analysis was used to investigate the effects of indoxyl sulfate as a continuous variable on incident PAD events, MACE, and death from any cause. Because the event number was relatively low, we avoided overfitting the model by selecting 4 clinically relevant covariates (age, current smoking, diabetes, and CVD) in the adjusted models. Effect modification of the association between serum indoxyl sulfate and PAD events by prespecified covariates (sex, albumin, nPCR, systolic BP, and the TC:HDL-C ratio) was tested by including multiplicative interaction terms in the multivariable model. A two-sided *p* value <0.05 was considered to be significant. Statistical analyses were performed using the computer software SPSS version 20.0 (SPSS Inc., Chicago, IL, USA) software.

## Figures and Tables

**Table 1:** Patient characteristics according to whether baseline PAD was present or not.

Characteristic	Overall	PAD at Baseline		*p* Value
	(*n* = 234)	No (*n* = 200)	Yes (*n* = 34)	
Age (years)	63 ± 13	62 ± 13	69 ± 11	0.007
Male sex, *n* (%)	128 (54.7%)	109 (54.5%)	19 (55.9%)	0.881
Current smoker, *n* (%)	27 (11.5%)	22 (11.0%)	5 (14.7%)	0.532
DM, *n* (%)	103 (44.0%)	74 (37.0%)	29 (85.3%)	<0.001
CVD, *n* (%)	76 (32.5%)	58 (29.0%)	18 (52.9%)	0.006
CAD, *n* (%)	64 (27.4%)	49 (24.5%)	15 (44.1%)	0.018
Stroke, *n* (%)	18 (7.7%)	13 (6.5%)	5 (14.7%)	0.097
Dialysis vintage (years)	2.4 (1.4–3.7)	2.4 (1.4–3.7)	2.6 (2.1–4.0)	0.109
BMI (kg/m^2^)	23.6 ± 3.8	23.6 ± 3.7	23.7 ± 4.1	0.849
ABI	1.06 ± 0.20	1.10 ± 0.16	0.81 ± 0.27	<0.001
Systolic BP (mmHg)	138 ± 20	138 ± 20	136 ± 19	0.533
RAASi, *n* (%)	75 (32.1%)	64 (32.0%)	11 (32.4%)	0.967
Anti-platelet, *n* (%)	52 (22.2%)	34 (17.0%)	18 (52.9%)	<0.001
Statin, *n* (%)	35 (15.0%)	22 (11.0%)	13 (38.2%)	<0.001
Kt/V	1.8 ± 0.3	1.8 ± 0.3	1.9 ± 0.3	0.318
URR (%)	78.0 ± 5.5	77.8 ± 5.7	78.6 ± 4.9	0.446
nPCR (g/kg/day)	1.1 ± 0.3	1.1 ± 0.3	1.0 ± 0.2	0.103
Hemoglobin (g/dL)	10.5 ± 1.2	10.5 ± 1.2	10.4 ± 1.3	0.893
Albumin (g/dL)	3.9 ± 0.4	3.9 ± 0.4	3.8 ± 0.3	0.170
TC (mg/dL)	158 (136–184)	159 (138–184)	152 (125–192)	0.446
HDL-C (mg/dL)	39 (31–49)	40 (31–50)	35 (27–46)	0.149
LDL-C (mg/dL)	89 (73–107)	91 (73–107)	81 (69–101)	0.142
Triglycerides (mg/dL)	123 (85–193)	126 (86–191)	112 (79–240)	0.942
TC:HDL-C	4.0 (3.0–5.3)	3.9 (3.0–5.4)	4.5 (2.9–5.3)	0.626
Fasting glucose (mg/dL)	98 (87–129)	96 (87–126)	110 (89–150)	0.112
Calcium (mg/dL)	9.3 (8.8–9.8)	9.4 (8.9–9.9)	9.1 (8.8–9.6)	0.140
Phosphate (mg/dL)	4.6 (3.7–5.7)	4.6 (3.8–5.7)	4.2 (3.1–5.7)	0.236
Intact PTH (pg/mL)	265 (126–431)	264 (117–429)	269 (156–436)	0.954
CRP (mg/L)	0.27 (0.12–0.63)	0.27 (0.11–0.59)	0.32 (0.21–0.79)	0.153
Indoxyl sulfate (μg/mL)	45.9 (29.9–62.6)	46.0 (29.7–62.9)	44.4 (30.4–62.2)	0.992

Abbreviations: ABI, ankle-brachial index; BMI, body mass index; BP, blood pressure; CAD, coronary artery disease; CRP, C-reactive protein; CVD, cardiovascular disease; DM, diabetes mellitus; HDL-C, high-density lipoprotein cholesterol; LDL-C, low-density lipoprotein cholesterol; nPCR, normalized protein catabolic rate; PAD, peripheral artery disease; PTH, parathyroid hormone; RAASi, renin-angiotensin-aldosterone system inhibitors; TC, total cholesterol; URR, urea reduction rate.

**Table 2:** Patient characteristics according to whether incident PAD was present or not.

Characteristic	Patients without Incident PAD (*n* = 163)	Patients with Incident PAD (*n* = 37)	*p* Value
Age (years)	61 ± 14	66 ± 10	0.045
Male sex, *n* (%)	86 (52.8%)	23 (62.2%)	0.300
Current smoker, *n* (%)	19 (11.7%)	3 (8.1%)	0.533
DM, *n* (%)	49 (30.1%)	25 (67.6%)	<0.001
CVD, *n* (%)	40 (24.5%)	18 (48.6%)	0.004
CAD, *n* (%)	33 (20.2%)	16 (43.2%)	0.003
Stroke, *n* (%)	10 (6.1%)	3 (8.1%)	0.660
Dialysis vintage (years)	2.4 (1.3–3.6)	2.4 (1.4–5.2)	0.876
BMI (kg/m^2^)	23.4 ± 3.8	24.2 ± 3.5	0.226
Systolic BP (mmHg)	138 ± 19	140 ± 22	0.650
RAASi, *n* (%)	50 (30.7%)	14 (37.8%)	0.399
Anti-platelet, *n* (%)	24 (14.7%)	10 (27.0%)	0.072
Statin, *n* (%)	15 (9.2%)	7 (18.9%)	0.088
Kt/V	1.8 ± 0.3	1.8 ± 0.3	0.612
URR (%)	77.9 ± 5.6	77.0 ± 6.0	0.393
nPCR (g/kg/day)	1.1 ± 0.3	1.1 ± 0.3	0.938
Hemoglobin (g/dL)	10.5 ± 1.3	10.4 ± 1.0	0.796
Albumin (g/dL)	3.9 ± 0.4	3.9 ± 0.3	0.731
TC (mg/dL)	158 (136–184)	162 (140–186)	0.763
HDL-C (mg/dL)	41 (32–51)	37 (29–44)	0.089
LDL-C (mg/dL)	90 (73–106)	95 (74–115)	0.607
Triglycerides (mg/dL)	126 (82–190)	121 (87–237)	0.352
TC:HDL-C	3.8 (2.9–5.4)	4.3 (3.3–5.4)	0.178
Fasting glucose (mg/dL)	95 (87–122)	110 (89–147)	0.193
Calcium (mg/dL)	9.4 (8.9–9.9)	9.2 (8.8–9.9)	0.486
Phosphate (mg/dL)	4.6 (3.8–5.7)	4.5 (3.8–5.8)	0.927
Intact PTH (pg/mL)	267 (116–422)	219 (116–450)	0.882
CRP (mg/L)	0.25 (0.12–0.58)	0.27 (0.10–0.61)	0.727
Indoxyl sulfate (μg/mL)	45.2 (28.5–61.7)	56.2 (40.9–69.0)	0.033

Abbreviations: BMI, body mass index; BP, blood pressure; CAD, coronary artery disease; CRP, C-reactive protein; CVD, cardiovascular disease; DM, diabetes mellitus; HDL-C, high-density lipoprotein cholesterol; LDL-C, low-density lipoprotein cholesterol; nPCR, normalized protein catabolic rate; PAD, peripheral artery disease; PTH, parathyroid hormone; RAASi, renin-angiotensin-aldosterone system inhibitors; TC, total cholesterol; URR, urea reduction rate.

**Table 3:** Association of risk factors and indoxyl sulfate with risk of incident PAD events.

	Model 1		Model 2	
	HR (95% CI)	*p* Value	HR (95% CI)	*p* Value
Age	1.05 (1.01–1.09)	0.006	1.05 (1.01–1.09)	0.008
Smoking	1.64 (0.47–5.72)	0.436	1.57 (0.46–5.42)	0.474
Diabetes	6.01 (2.84–12.71)	<0.001	6.46 (2.97–14.06)	<0.001
Cardiovascular disease	2.34 (1.12–4.90)	0.024	2.53 (1.23–5.18)	0.011
Indoxyl sulfate by tertiles^a^				
Lower tertile	1.00	–	–	–
Middle tertile	2.03 (0.80–5.18)	0.138	–	–
Upper tertile	3.20 (1.26–8.12)	0.014	–	–
Indoxyl sulfate, per 10 μg/mL	–	–	1.19 (1.05–1.35)	0.006

Abbreviations: CI, confidence interval; HR, hazard ratio. Model 1 was constructed based on inclusion of indoxyl sulfate as a categorical variable. Model 2 was constructed based on inclusion of indoxyl sulfate as a continuous variable. ^a^Lower tertile: 0.37–34.4 μg/mL; Middle tertile: 34.9–57.9 μg/mL; Upper tertile: 57.9–141.4 μg/mL.

**Table 4:** Cox regression hazard ratios with 95% confidence intervals for three outcomes.

Predictor	PAD	MACE	Mortality
Indoxyl sulfate (10 μg/mL increase)	1.19 (1.05–1.35) ^a^	1.00 (0.90–1.12)	0.98 (0.90–1.07)
BMI (kg/m^2^)	1.04 (0.94–1.14)	1.05 (0.97–1.14)	0.96 (0.90–1.02)
Systolic BP (10 mmHg increase)	1.08 (0.90–1.29)	1.17 (1.00–1.36)	1.04 (0.93–1.17)
TC (10 mg/dL increase)	1.04 (0.96–1.13)	1.03 (0.96–1.11)	1.01 (0.95–1.07)
HDL-C (10 mg/dL increase)	0.94 (0.75–1.17)	0.91 (0.76–1.11)	0.92 (0.79–1.07)
LDL-C (10 mg/dL increase)	1.08 (0.98–1.19)	1.10 (1.00–1.21) ^b^	1.03 (0.96–1.11)
Triglycerides (10 mg/dL increase)	1.00 (0.97–1.03)	1.00 (0.97–1.02)	1.01 (0.99–1.03)
TC:HDL-C	1.05 (0.93–1.18)	1.01 (0.90–1.13)	0.99 (0.90–1.10)
Calcium (mg/dL)	1.04 (0.68–1.61)	1.61 (1.15–2.27) ^a^	1.46 (1.12–1.89) ^a^
Phosphate (mg/dL)	1.06 (0.86–1.31)	0.99 (0.81–1.22)	0.93 (0.78–1.10)
Ln CRP (mg/L)	0.91 (0.67–1.24)	1.06 (0.82–1.37)	1.31 (1.10–1.56) ^a^

Abbreviations: BMI, body mass index; BP, blood pressure; CRP, C-reactive protein; HDL-C, high-density lipoprotein cholesterol; LDL-C, low-density lipoprotein cholesterol; Ln, natural logarithm; MACE, major adverse cardiovascular event; PAD, peripheral artery disease; TC, total cholesterol. Separate models for each candidate risk factor; each model was adjusted for age, smoking, diabetes, and cardiovascular disease. ^a^ *p* < 0.01, ^b^ *p* < 0.05.
